# Regimen comprising GLP-1 receptor agonist and basal insulin can decrease the effect of food on glycemic variability compared to a pre-mixed insulin regimen

**DOI:** 10.1186/s40001-022-00892-9

**Published:** 2022-12-03

**Authors:** Yi-Hsuan Lin, Chia-Hung Lin, Yu-Yao Huang, Hsin-Yun Chen, An-Shun Tai, Shih-Chen Fu, Sheng-Hwu Hsieh, Jui-Hung Sun, Szu-Tah Chen, Sheng-Hsuan Lin

**Affiliations:** 1 Division of Endocrinology and Metabolism, Department of Internal Medicine, Chang Gung Memorial Hospital, Linkou branch, Taoyuan, Taiwan; 2 Department of Chinese Medicine, College of Medicine, Chang Gung University, Taoyuan, Taiwan; 3 Department of Medical Nutrition Therapy, Chang Gung Memorial Hospital, Linkou branch, Taoyuan, Taiwan; 4 Institute of Statistics, National Chiao Tung University, Hsinchu, Taiwan; 5 Department of Biological Science and Techonology, National Yang Ming Chiao Tung University, Hsinchu, Taiwan

**Keywords:** Continuous glucose monitoring, Glucose variability, Pre-mixed insulin, GLP-1 receptor agonist, Diet

## Abstract

**Background:**

Increasing evidence suggests that glucagon-like peptide 1 (GLP-1) receptor agonists (RA) can stabilize glycemic variability (GV) and interfere with eating behavior. This study compared the impact of insulin, GLP-1 RA, and dietary components on GV using professional continuous glucose monitoring (CGM).

**Methods:**

Patients with type 2 diabetes underwent CGM before and after switching from a twice-daily pre-mixed insulin treatment regimen to a GLP-1 RA (liraglutide) plus basal insulin regimen. The dietary components were recorded and analyzed by a certified dietitian. The interactions between the medical regimen, GV indices, and nutrient components were analyzed.

**Results:**

Sixteen patients with type 2 diabetes were enrolled in this study. No significant differences in the diet components and total calorie intake between the two regimens were found. Under the pre-mixed insulin regimen, for increase in carbohydrate intake ratio, mean amplitude of glucose excursion (MAGE) and standard deviation (SD) increased; in contrast, under the new regimen, for increase in fat intake ratio, MAGE and SD decreased, while when the protein intake ratio increased, the coefficient of variation (CV) decreased. The impact of the food intake ratio on GV indices disappeared under the GLP-1 RA regimen. After switching to the GLP-1 RA regimen, the median MAGE, SD, and CV values decreased significantly. However, the significant difference in GV between the two regimens decreased during the daytime.

**Conclusion:**

A GLP-1 RA plus basal insulin regimen can stabilize GV better than a regimen of twice-daily pre-mixed insulin, especially in the daytime, and can diminish the effect of food components on GV.

**Supplementary Information:**

The online version contains supplementary material available at 10.1186/s40001-022-00892-9.

## Background

In patients with diabetes mellitus, the target of blood sugar control is to maintain the mean glucose level, which is presented as glycated hemoglobin A1c (HbA1c) along with the glycemic variability (GV) [1]. High GV increases oxidative stress and inflammation [2, 3], which could be the possible etiologies of cardiovascular complications in diabetes mellitus [4]. Owing to technological advancements, the continuous glucose monitoring (CGM) system can evaluate the glucose level in the interstitial fluid, which is representative of the blood glucose level [5]. Furthermore, by evaluating the fluctuation in glucose levels and calculating the GV indices, physicians can adjust medication in time and further improve patient outcomes [6, 7]. Food intake plays an important role in GV. In both type 1 and type 2 diabetes, a low carbohydrate diet for 3 months can decrease GV and antidiabetic medication requirement [8, 9]. Further, the carbohydrate (CHO), protein (PRO), and fat intake have an impact on glucose control and cardiovascular risk factors in patients with diabetes mellitus [10].

Various categories of antidiabetic drugs are being used, and as a new class of antidiabetic agents, namely, glucagon-like peptide 1 (GLP-1) receptor agonists (RA), can decrease appetite and body weight and increase insulin sensitivity. In addition, GLP-1 RA provides cardiovascular protection [11, 12]. Bajaj et al. showed that long-acting GLP-1 RA combined with basal insulin had a lower GV than basal-bolus insulin, pre-mixed insulin, or basal-bolus insulin combined with oral drugs [13]. However, insulin is the cornerstone of glucose control, especially in patients with type 2 diabetes and beta-cell function failure [14]. However, use of insulin increases the risk of hypoglycemia [13].

Although several studies on GLP-1 RA exist, no research has investigated the effect of GLP-1 RA on diet components and GV compared with that of the pre-mixed insulin regimen in the same patients.

Therefore, to evaluate the interaction between GV, food intake, and diabetes mellitus treatment regimen, we enrolled 16 patients with type 2 diabetes who were under a pre-mixed insulin regimen and applied the first CGM. Additionally, the diet components were recorded. We then changed the treatment regimen to GLP-1 RA combined with basal insulin and applied a second CGM. We compared GV and dietary components before and after the treatment shift. This pilot study aimed to analyze the effects of insulin and GLP-1 RA on GV and food intake.

## Methods

### Participants

Sixteen patients (eight females and eight males) with type 2 diabetes who were treated with pre-mixed insulin combined with other oral antidiabetic drugs (OADs) were enrolled. Patients who were independent and cooperated to receive CGM and recorded their diet log by themselves or with the help of a family member were included. They received their first CGM at baseline. We then calculated the dose of insulin to be basal insulin in the pre-mixed insulin regimen and then shifted to insulin glargine U300 with the same dose combined with liraglutide, and titrated or tapered the doses according to the patients’ response. The second CGM was performed three months later after shifting to basal insulin (insulin glargine U300) combined with a GLP-1 RA (liraglutide) regimen when the patients’ sugar levels were relatively stable. Eleven patients were injected with liraglutide in the morning and five patients were injected in the evening, and all patients received insulin glargine U300 in the morning.

Physical activity and dietary habits were the patients’ routines. The exclusion criteria were (a) presence of any severe cardiovascular disorder, (b) history of influenza infection, (c) autoimmune disease or metabolic disorders, (d) drug or alcohol abuse, and (e) participation in another clinical investigation study. The medical records of all participants were retrospectively reviewed. This study was approved by the Institutional Review Board (IRB) and the ethics committee of Chang Gung Memorial Hospital (CGMH) (IRB No. 201701492B0). The IRB waived the requirement for obtaining informed consent , as this study was retrospective and the decision making of clinical physicians was not interfered with, at that time. The confidentiality of the research subjects was maintained according to the requirements of the IRB of CGMH (Taipei, Taiwan).

### Diet records

The participants and their family members were educated on how to record the components of all the food items they consumed by a certified dietitian. The amount and type of food, fruits, snacks, beverages, and desserts, and the method of preparation was recorded. The details of nutrient elements, including the weight and calories of CHO, PRO, and fats, were analyzed by the dietitian after CGM completion, and the ratio of calories from each element to the total daily calories was calculated. We included diet analysis results on the days in which the patients had an intact 24-h CGM record.

### Glucose monitoring

CGM was conducted using iPro™2 Professional CGM (Medtronic, Inc., Northridge, CA, USA). The CGM sensor needle was pierced into the subcutaneous tissue of the abdomen, buttocks, or arms of the participants. All patients underwent 24-h monitoring for approximately 6 d at home and we included data on the days in which the patients had intact 24-h CGM record. The sensor detects the glucose level in the interstitial fluid every 10 s and outputs an average value every 5 min. A time lag for glucose level change in the interstitial fluid existed as diffusion of glucose from the serum to the interstitial space takes time [15]. Therefore, patients were required to input capillary blood sugar levels four times a day to correct the output value. CGM could recognize interstitial fluid glucose levels from 40 to 400 mg/dL. If the values were out of this range, the CGM output value would be 40 or 400 mg/dL.

The HbA1c level was measured within 3 months before CGM and again after the first and second CGM.

### Glucose variability indices measures

The collected data were downloaded using CareLink™ iPro software (Medtronic, Inc., Northridge, CA, USA) after the completion of this study. Several parameters were used to demonstrate the GV level, including standard deviation (SD) [16], percentage coefficient of variation (%CV) [16], and mean amplitude of glucose excursion (MAGE) [16]. The time spent with glucose levels within 70–180 mg/dL was considered the time in range (TIR), which was approximately half the self-monitoring of blood glucose (SMBG) recorded within this range. Thus, the HbA1c level was approximately 7% [17]. The HbA1c level can be expressed as a percentage, which means that the proportion of the glucose level within this range accounts for the total reading time. The area under the curve (AUC) of glucose levels > 180 mg/dL (AUC_180_) and < 70 mg/dL (AUC_70_) was represented as hyperglycemic and hypoglycemic periods, respectively. AUC_t_ and AUC_n_ represent the total and normal (70–180 mg/dL) AUCs for glucose levels, respectively [18]. The low blood glucose index (LBGI) and high blood glucose index (HBGI) were calculated based on glucose levels and represented the risk of hypoglycemia and hyperglycemia, respectively [19]. The M-value was calculated to evaluate GV [19]. Continuous overlapping net glycemic action (CONGA) was used as an indicator for the evaluation of blood glucose fluctuations in a relatively short period. It assesses intraday GV at different set intervals [19]. For instance, CONGA_4_ represents the standard deviation of the blood glucose level every 4 h compared to that recorded at the previous interval.

### Statistical analysis

All statistical analyses were performed using Statistical Analyses Package Program SPSS Statistics for Windows (version 26.0; SPSS, Inc., Chicago, IL, USA). Differences in continuous variables between the two regimens were calculated using the Wilcoxon signed-rank test. Nominal variables were analyzed using the McNemar’s Chi-square test. A linear mixed model was used for the two continuous variants to predict the response of a single variable. The relationship between two variables was measured to determine the strength of each variable using Spearman’s correlation coefficient. Statistical significance was set at *p* < 0.05.

## Results

All patients completed CGM under pre-mixed insulin and GLP-1 RA plus basal insulin regimens. The average interval between the first and second CGM was 67.5 d. All participants were aged between 44.2 and 85.5 years, and the average duration of diabetes mellitus was 10.8 years. The demographic characteristics of all participants are summarized in Table 1. The most frequently combined OAD with a pre-mixed insulin regimen and GLP-1 RA was metformin (56.3% and 68.8%, respectively).

**Table 1:** Demographic and diet characteristics of participants

	Pre-mix insulin regimen (*n* = 16)	GLP-1 RA added on basal insulin regimen (*n* = 16)	*p*
Age (y)	59.8 [50.3, 70.1]	–	–
Sex, male (n,%)	8(50)	–	–
Body weight (kg)	71.5 [61.8, 84.5]	71.5 [61.8, 84.5]	0.500
Height (cm)	164.8 [150.5, 169.8]	–	–
BMI (kg/m^2^)	27.0 [25.0, 30.9]	–	–
Duration of disease (y)	10 [6, 14.5]	–	–
HbA1c (%, mmol/mol) before CGM	9.5 [8.7, 10.9] 80.3 [71.6, 95.6]	9.4 [8.3, 9.9] 78.7 [67.5, 85.0]	0.099
HbA1c (%, mmol/mol) after CGM	9.4 [8.3, 9.9] 78.7 [67.5, 85.0]	9.0 [7.8, 10.1] 74.9 [61.5, 86.6]	0.767
Difference of HbA1c (%, mmol/mol) before and after CGM	− 0.7 [− 1.4, 0.2] − 7.7 [− 15.3, 2.2]	0 [− 0.3, 0.3] 0 [− 3.3, 3.0]	0.125
Total daily insulin dose (U)	50.0 [45.3, 55.8]	25.0 [21.0, 33.0]	< 0.001^a^
Total daily insulin dose/BW (U/kg)	0.61 [0.52, 0.82]	0.36 [0.27, 0.43]	< 0.001^a^
Basal daily insulin dose (U)	35.0 [30.4, 38.9]	25.0 [21.0, 33.0]	0.001^a^
Bolus daily insulin dose(U)	14.5 [13.1, 16.4]	0.0 [0.0, 0.0]	< 0.001^a^
Combined with OAD type			
Metformin (N, %)	9 (56.3)	11(68.8)	0.424
Sitagliptin (N, %)	4 (25)	0 (0)	0.012^a^
Dapagliflozin (N, %)	1 (6.3)	0 (0)	< 0.001^a^
Acarbose (N, %)	2 (12.5)	2 (12.5)	0.004^a^
Pioglitazone (N, %)	0 (0)	1 (6.3)	< 0.001^a^
Glimepiride (N, %)	0 (0)	1 (6.3)	< 0.001^a^
Nutrient composition (per day)			
CHO (%)	50.5 [46.0, 54.8]	50.5 [42.0, 53.8]	0.605
CHO (g)	158.4 [143.0, 175.6]	143.3 [118.6, 168.3]	0.163
PRO (%)	14.7 [13.1, 16.0]	15.1 [13.6, 18.0]	0.148
PRO (g)	48.4 [34.4, 60.7]	41.5 [32.2, 58.0]	0.569
Fat (%)	34.6 [32.1, 38.4]	35.7 [31.6, 40.0]	0.717
Fat (g)	48.9 [36.7, 64.1]	45.3 [30.4, 63.3]	0.289
Average calories per day (kcal)	1228.0 [1038.6, 1499.5]	1231.0 [879.5, 1412.6]	0.179
Calories/ body weight (kcal/kg)	16.6 [14.1, 21.3]	17.0 [12.6, 19.9]	0.215
Carbohydrate (g) / body weight (kg)	2.01 [1.88, 2.56]	2.05 [1.58, 2.39]	0.215
PRO (g) / body weight (kg)	0.58 [0.53, 0.86]	0.61 [0.55, 0.79]	0.796
Fat (g) / body weight (kg)	0.68 [0.53, 0.88]	0.65 [0.43, 0.80]	0.469

Age, body weight, height, body mass index, duration of disease, HbA1c before/after CGM, insulin dose, and nutrient composition are presented as the median [Q1, Q3]. Continuous variants were analyzed by Wilcoxon singed-rank test and nominal variants were analyzed by McNemar’s Chi-square test

*GLP-1 RA* Glucagon-like peptide 1 receptor agonist; *BMI* Body mass index; *HbA1c* Glycated hemoglobin *A1c CGM* Continuous glucose monitoring; *OAD* Oral antidiabetic drug; *CHO* Carbohydrate; *PRO* Protein

"a denotes p value < 0.05"

Participants required higher total daily insulin doses and more basal insulin doses under the pre-mixed insulin regimen than under the GLP-1 RA combined with basal insulin regimen. The total daily median insulin dose was 50 U in the pre-mixed insulin regimen and 25 U in the GLP-1 RA combined with basal insulin regimen. The basal insulin dose used was higher in the pre-mixed insulin regimen. Regarding nutrient composition, although no statistical significance was found, participants consumed more calories under the pre-mixed insulin regimen than under the GLP-1 RA combined with basal insulin regimen. With regard to nutrient ingredients, no difference in CHO, PRO, and fat intake ratios between the two regimens was found.

Whole-day SD, CV, MAGE, CONGA_2_, and CONGA_4_ levels were lower in the GLP-1 RA combined with basal insulin regimen than in the pre-mixed insulin regimen. However, no difference in nocturnal glucose variability indices between the two regimens was found (Table 2). Under the pre-mixed insulin regimen, for every 1% increase in calorie ratio of CHO intake, MAGE and SD increased by 2.699 mg/dL and 1.324 mg/dL, respectively. For every 1% increase in calorie ratio of fat intake, MAGE and SD decreased by 3.487 mg/dL and 1.595 mg/dL, respectively. For every 1% increase in calorie ratio of PRO intake, CV decreased by 0.021% (Table 3). Briefly, increased CHO intake ratio was moderately and positively correlated with increased SD and CV (r = 0.366 and 0.420, respectively, *p* < 0.05). However, increased PRO and fat intake ratios moderately and negatively correlated with reduced CV (r = –0.423 and –0.363, respectively; *p* < 0.05). Moreover, MAGE, SD, and CV had a moderate positive correlation with age and the duration of diabetes mellitus (Additional file 1: Table S1).

**Table 2:** Results of computerized glycemic variability index

	Pre-mix insulin usage (*N* = 16)	GLP-1 agonist added on basal insulin usage (*n* = 16)	*p*
All day period (0:00–24:00)			
SD	43.9 [34.4, 60.6]	36.0 [27.7, 47.5]	0.017^a^
CV	0.24 [0.21, 0.29]	0.20 [0.15, 0.26]	0.046^a^
MAGE	114.2 [91.4, 151.4]	78.7 [66.8, 124.8]	0.006^a^
AUCt	50869.2 [44430.2, 66563.7]	53484.3 [41642.7, 59404.7]	0.918
AUC180	31850.4 [15978.1, 55968.9]	34434.3 [12336.0, 46503.3]	0.535
AUCn	18672.2 [9534.4, 28527.5]	19419.5 [13836.7, 28965.2]	0.408
AUC70	0.0 [0.0, 175.0]	0.0 [0.0, 0.0]	0.674
LBGI	0.07 [0.00, 0.41]	0.00 [0.00, 0.18]	0.463
HBGI	9.68 [5.41, 20.28]	9.46 [4.14, 14.48]	0.535
M-value	23.47 [13.12, 45.15]	18.45 [10.49, 27.86]	0.088
CONGA1	28.07 [23.33, 39.29]	23.25 [19.93, 34.58]	0.079
CONGA2	42.80 [35.88, 56.29]	36.45 [31.46, 54.49]	0.049^a^
CONGA4	56.94 [45.81, 70.33]	50.49 [38.80, 62.95]	0.020^a^
TIR	39.0 [24.1, 71.7]	52.6 [31.4, 77.6]	0.305
Duration above upper limit (%)	61.0 [27.0, 76.0]	47.4 [19.9, 68.6]	0.352
Duration within limits (%)	39.0 [24.1, 71.7]	52.6 [31.4, 77.6]	0.453
Duration below lower limit (%)	0.0 [0.0, 0.6]	0.0 [0.0, 0.0]	0.624
Nocturnal period (00:00–06:00)			
SD	14.8 [9.3, 20.2]	11.5 [9.39, 19.9]	0.605
CV	0.09 [0.08, 0.13]	0.07 [0.06, 0.13]	0.796
MAGE	34.9 [23.1, 50.4]	32.9 [25.1, 60.0]	0.642
AUCt	11771.3 [8691.5, 14551.0]	11015.0 [8383.6, 12973.3]	0.569
AUC180	3822.2 [260.1, 12524.9]	2262.3 [13.6, 6654.3]	0.363
AUCn	6176.7 [2794.0, 8419.7]	7392.8 [5662.3, 8730.4]	0.278
AUC70	0.0 [0.0, 0.0]	0.0 [0.0, 0.0]	0.715
LBGI	0.09 [0.00, 0.59]	0.00 [0.00, 0.51]	0.552
HBGI	6.24 [1.32, 14.17]	4.31 [0.86, 8.44]	0.379
M-value	8.96 [5.01, 22.66]	7.71 [3.32, 15.82]	0.408
CONGA1	13.20 [9.52, 16.57]	10.61 [8.53, 13.21]	0.438
CONGA2	14.12 [11.61, 19.84]	12.58 [10.17, 16.80]	0.234
CONGA4	11.64 [9.15, 13.36]	9.87 [8.08, 14.26]	0.642

Each glycemic variability index is presented as the median [Q1, Q3]. Continuous variants were analyzed by Wilconxon singed-rank test

*SD* standard deviation; *CV* coefficient of variation; *MAGE* mean amplitude of glycemic excursions; *AUC* area under curve; *LBGI* low blood glucose index; *HBGI* high blood glucose index; *M-value* weighted average of glucose value; *CONGA* continuous overlapping net glycemic action; *TIR* time in range

^a^denotes *p* value < 0.05

**Table 3:** Impact of nutrient components on MAGE, SD and CV

Parameter	MAGE (mg/dl)								
	Total person times (*n* = 32)			Pre-mix insulin regimen (*n* = 16)			GLP-1 RA on basal insulin regimen (*n* = 16)		
Variable	β	95% CI of β	*P*	β	95% CI of β	*p*	β	95% CI of β	*p*
CHO (%)	1.141	(− 0.481,2.763)	0.168	2.699	(0.067,5.332)	0.045^a^	0.918	(− 1.567, 3.404)	0.441
Pro (%)	− 6.494	(− 12.124, -0.865)	0.023^a^	− 3.462	(− 14.797,7.873)	0.523	− 2.267	(− 11.893, 7.358)	0.621
Fat (%)	− 1.092	(− 3.013, 0.827)	0.264	− 3.487	(− 6.529, -0.446)	0.028^a^	− 1.076	(− 4.028, 1.876)	0.448
CHO (gram/kg)	14.80	(− 11.941,41.546)	0.278	16.77	(− 28.008,61.556)	0.435	11.49	(− 30.454,53.428)	0.566
Pro (gram/kg)	− 51.39	(− 123.139,20.357)	0.160	− 47.77	(− 159.053,63.518)	0.373	− 18.49	(− 123.15,86.172)	0.711
Fat (gram/kg)	− 26.80	(− 82.136,28.523)	0.342	− 67.82	(− 146.583,10.939)	0.086	− 23.13	(− 107.945, 61.689)	0.568
Parameter	SD (mg/dl)								
CHO (%)	0.440	(− 0.165,1.045)	0.154	1.324	(0.409,2.239)	0.008^a^	0.266	(− 0.704,1.235)	0.566
PRO (%)	− 2.768	(− 4.786,-0.750)	0.007v	− 2.854	(− 7.023,1.315)	0.164	− 1.155	(− 4.849,2.539)	0.513
Fat (%)	− 0.369	(− 1.085,0.347)	0.312	− 1.595	(− 2.677–0.513)	0.007^a^	− 0.263	(− 1.419,0.892)	0.632
CHO (gram/kg)	4.874	(− 5.153,14.901)	0.340	8.475	(− 8.677,25.627)	0.307	4.403	(− 11.806,20.612)	0.570
PRO (gram/kg)	− 17.00	(− 44.015,10.019)	0.217	− 21.63	(− 64.482,21.213)	0.297	− 3.635	(− 44.228,36.959)	0.851
Fat (gram/kg)	− 5.428	(− 26.362,15.469)	0.610	− 25.17	(− 56.163,5.821)	0.103	− 4.297	(− 37.377,28.782)	0.785
Parameter	CV (%)								
CHO (%)	0.003	(0.0007,0.006)	0.011^a^	0.004	(0,0.009)	0.061	0.003	(− 0.001,0.006)	0.120
PRO (%)	− 0.013	(− 0.023,-0.003)	0.013^a^	− 0.021	(− 0.036,-0.006)	0.009^a^	− 0.004	(− 0.019,0.011)	0.566
Fat (%)	− 0.004	(− 0.007,-0.0002)	0.032^a^	− 0.004	(− 0.01,0.002)	0.158	− 0.004	(− 0.008,0.001)	0.095
CHO (gram/kg)	0.006	(− 0.041,0.052)	0.806	0.001	(− 0.075,0.078)	0.971	− 0.002	(− 0.593,0.553)	0.941
PRO (gram/kg)	− 0.134	(− 0.243,-0.025)	0.016^a^	− 0.187	(− 0.346,-0.029)	0.023^a^	− 0.1	(− 0.254,0.054)	0.186
Fat (gram/kg)	− 0.097	(− 0.182,-0.012)	0.025^a^	− 0.113	(− 0.245,0.018)	0.085	− 0.105	(− 0.225,0.014)	0.080

Linear mixed model analysis with β as standardized coefficient

*MAGE* mean amplitude of glycemic excursions; *SD* standard deviation; *CV* coefficient of variation; *CHO* carbohydrate; *PRO* protein

^a^denotes *p* value < 0.05

**Fig. 1:**
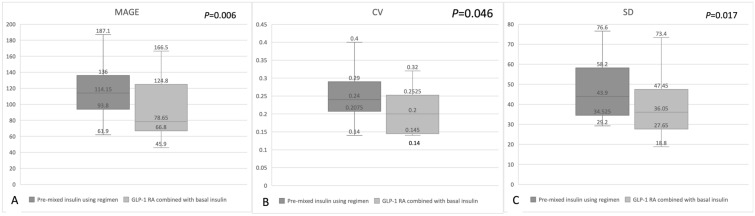
Changes in **A** MAGE **B** CV **C** SD from the pre-mixed insulin regimen to the GLP-1 RA combined with basal insulin regimen. The data are presented as median [Q1, Q3]

**Fig. 2:**
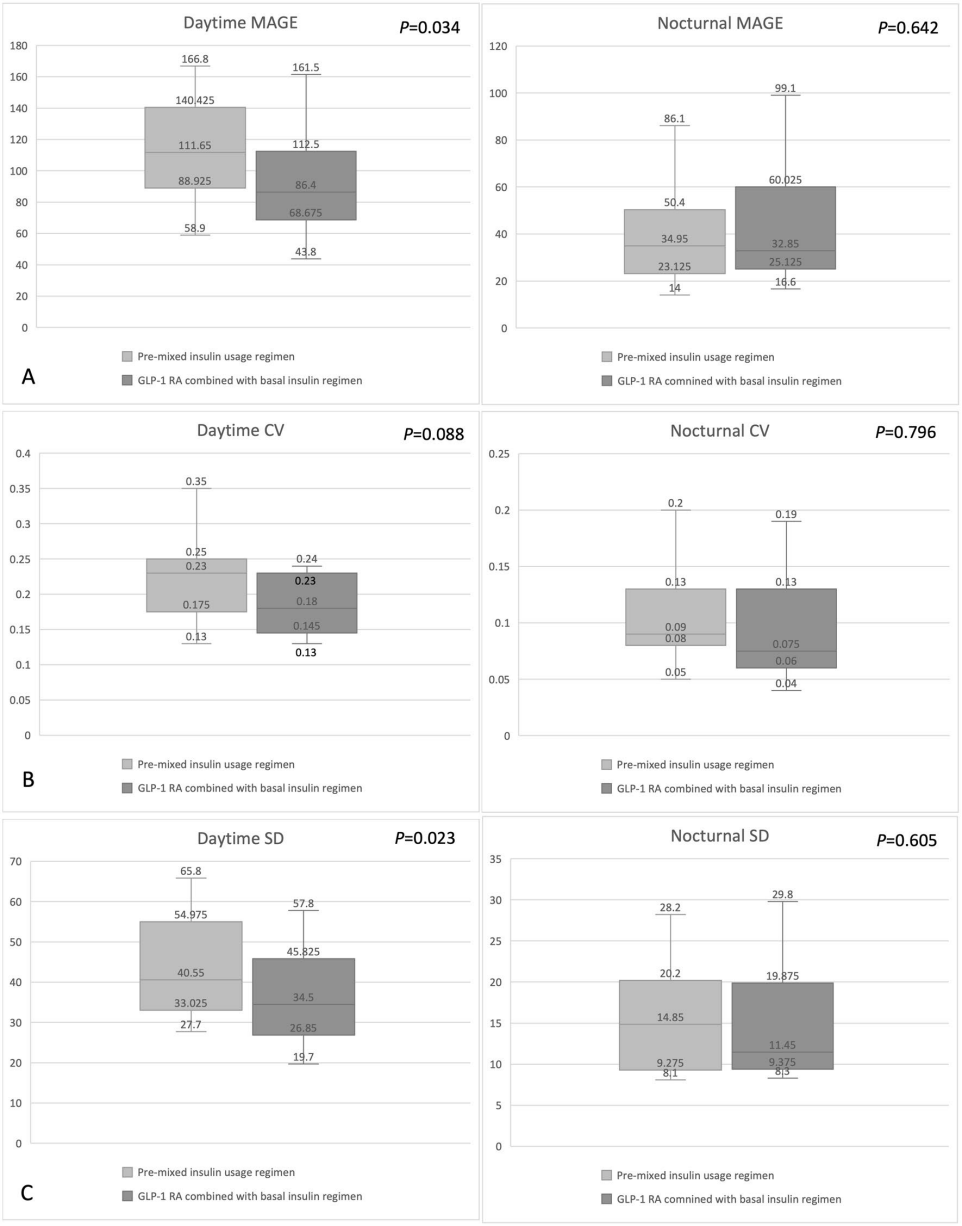
Changes in daytime and nocturnal **A** MAGE **B** CV **C** SD from the pre-mixed insulin regimen to the GLP-1 receptor agonist combined with basal insulin regimen. The data are presented as median [Q1, Q3]

**Fig. 3:**
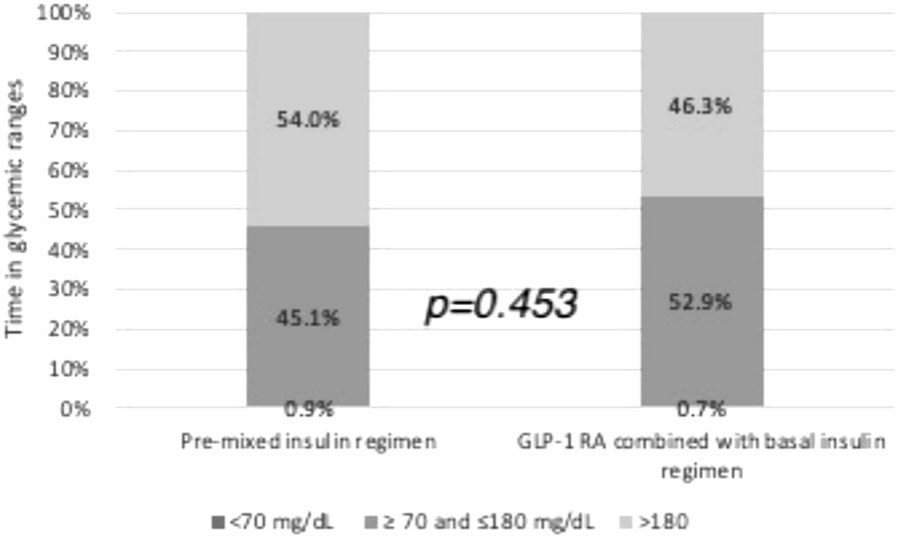
Changes in the time spent (%) in glucose ranges from the pre-mixed insulin regimen to the GLP-1 RA combined with basal insulin regimen

**Fig. 4:**
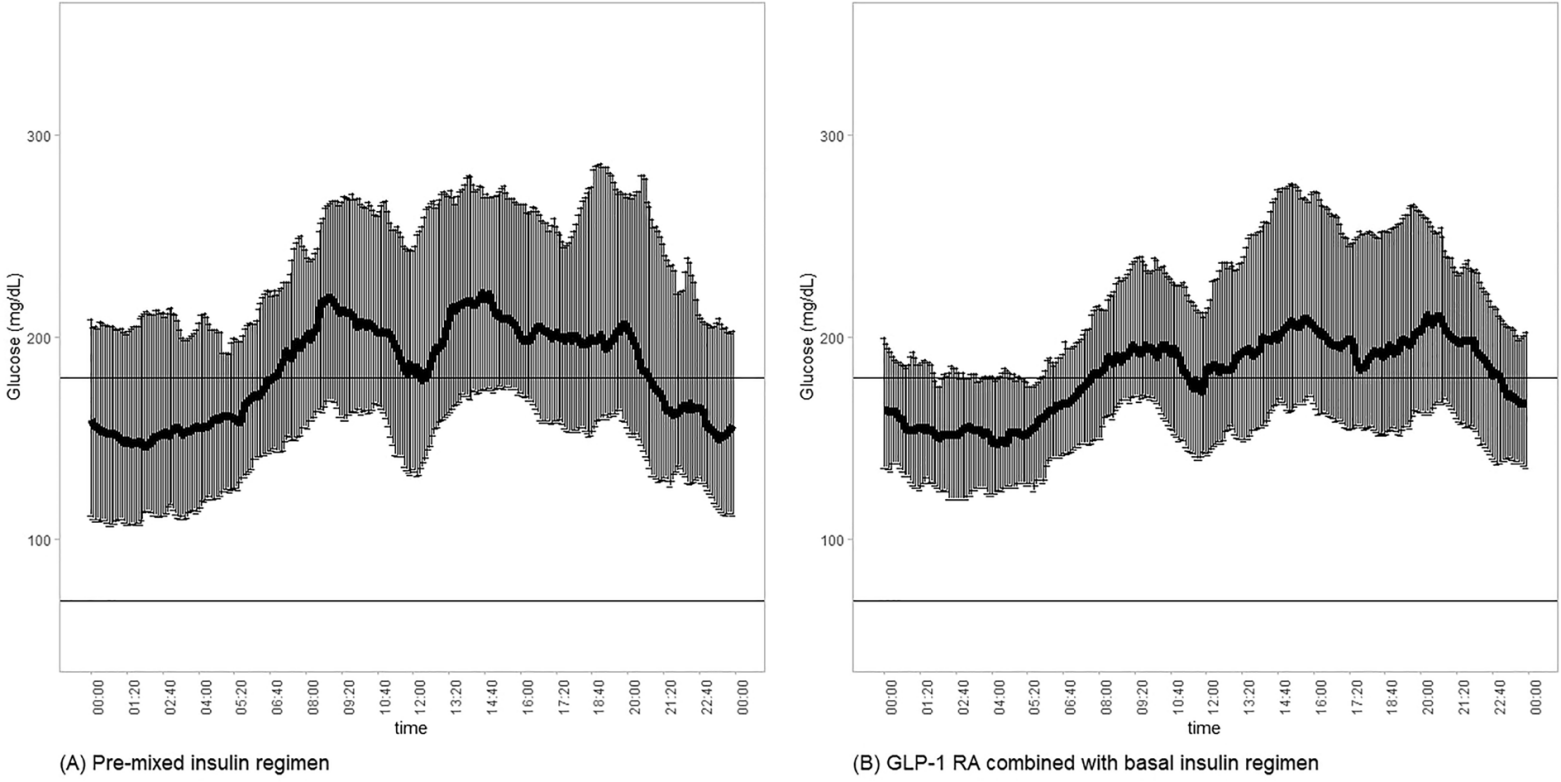
Time course of mean blood glucose variations in individual subjects. **A** Participants treated with the pre-mixed insulin regimen and **B** participants treated with the GLP-1 RA combined with basal insulin regimen. The data are presented as mean ± 95% confidence intervals

After the participants switched from a pre-mixed insulin regimen to a GLP-1 RA combined with basal insulin regimen, the median MAGE, SD, and CV decreased significantly from 114.2 to 78.7 mg/dL, from 43.9 to 36.0 mg/dL, and from 0.24 to 0.20%, respectively (Table 2 and Fig. 1). However, the impact of food intake on GV was only observed during the daytime. No significant reduction in nocturnal MAGE, SD, or CV was found (Fig. 2). Although not statistically significant, the TIR ratio increased from 45.1 to 52.9% after the participants changed to the GLP-1 RA combined with basal insulin regimen (Fig. 3). The correlation between the calorie ratio of CHO and TIR was − 0.224 and − 0.044 in the pre-mixed insulin regimen and basal insulin plus GLP-1 RA regimen, respectively. The results were not statistically significant. The time course of the mean blood glucose variations in individual subjects in the two groups is shown in Fig. 4.

## Discussion

The current pilot study analyzed the correlation between diet components, GV, and antidiabetic regimens of pre-mixed insulin or GLP-1 RA plus basal insulin using CGM and diet records.

GLP-1 RA can reportedly improve blood glucose control by increasing insulin secretion and insulin sensitivity and decreasing appetite and body weight [20]. However, insulin is the most effective blood glucose-lowering agent. Therefore, for patients with hyperglycemic urgency or very poorly controlled diabetes mellitus, insulin is the first-line therapy [21], although it increases the risk of hypoglycemia. Insulin is a regulator of appetite [22] and can decrease appetite. However, their physiological metabolism leads to increased body weight [23, 24], which, in turn, leads to higher GV [25].

Liraglutide, a once-daily GLP-1 RA, was approved for use as an antidiabetic and body weight control medication by the US Food and Drug Administration [26]. The drug helps in delaying gastric emptying and suppresses appetite by inhibiting the neuronal pathway. In addition, the drug controls blood sugar by increasing insulin secretion and insulin sensitivity and decreasing hepatic glucose production [27]. In contrast, central administration of insulin inhibits appetite and causes weight loss [22]. However, insulin therapy in diabetes causes body weight and fat mass gain owing to its anabolic effect and dietary compensation for hypoglycemia [28, 29]. In this study, no statistically significant difference was found in the nutrient components and total calorie intake every day between the two regimens, but a lower daily calorie intake was observed under the pre-mixed insulin regimen; however, evaluating significance of the difference is difficult, owing to the small sample size. In addition, insulin has an anorexic effect, although central insulin resistance may decrease appetite [29]. Liraglutide with basal insulin regimen was associated with decreased total daily insulin dose and basal insulin dose (from 35 to 25 median units basal insulin, *p* < 0.001 for both) compared with the pre-mixed insulin regimen. Carris et al. reviewed several studies and showed that liraglutide combined with basal insulin can reduce the basal insulin dose, body weight, and risk of hypoglycemia [29]. Adjustments in OAD were made after switching to basal insulin combined with the GLP-1 RA regimen. Given the limitations of the drug mechanism (both incretin-based therapy with DPP-4 inhibitors and GLP-1 RA) and health insurance in Taiwan (cannot use GLP-1 RA and SGLT2 inhibitor simultaneously), the OAD must be adjusted while shifting regimens. However, the results still represent real-world conditions and provide suggestions for clinical physicians.

Nutrient components were found to interfere with GV, especially during the pre-mixed insulin regimen. An increase in CHO intake ratio increased GV, whereas an increase in PRO and fat intake ratios decreased GV. Only a few studies have analyzed the correlation between nutrients and GV. Tay et al. showed that, in patients with type 2 diabetes, a low-CHO diet can reduce CONGA_1_ and diabetes medication requirements compared with a high-CHO diet [9]. Thomsen et al. tried to replace the conventional diabetes mellitus diet with a CHO-reduced high-PRO diet and showed a 36–45% reduction in GV indices [30]. Moreover, previous studies have demonstrated that the higher the PRO percentage intake, the lower the MAGE level [31]. Mori et al. further demonstrated that a low-CHO and high-monounsaturated fatty acid liquid diet could stabilize GV and reduce HbA1c levels compared to a high-CHO liquid diet [32]. Furthermore, SD is reportedly lower with the basal insulin plus GLP-1 RA (exenatide or liraglutide) regimen than with the pre-mixed insulin regimen [13], consistent with the findings of the present study. Moreover, when combined with basal insulin or with multiple daily insulin injections, liraglutide could increase TIR, regardless of daytime or nighttime [33]. In contrast, even when not combined with GLP-1 RA, basal insulin can decrease GV in comparison to pre-mixed insulin [34]. Taken together, macro-nutritional components interfere with GV, and both GLP-1 RA and basal insulin can lead to a decline in GV.

Both age and duration of diabetes mellitus had a moderately negative correlation with MAGE, CV, and SD, indicating that the longer the duration of diabetes mellitus and the older the patient, the higher the GV index. This result is consistent with those of many previous reports. Tong et al. showed that patients with type 2 diabetes and HbA1c levels > 7% had longer diabetes duration and higher GV indices [35]. Furthermore, Gude et al. [36] noted an increase in GV indices with age. In the Diabetes Outcomes in Veterans Study, 204 patients with type 2 diabetes receiving insulin treatment were included and underwent 8-week self-monitoring of blood glucose levels. The follow-up results showed that older participants and those with a longer duration of insulin treatment had higher GV [37]. However, Noyes et al. reported the opposite finding in a case–control study involving 10,130 participants with type 2 diabetes; the group revealed that younger participants had higher HbA1c variability [38]. Because of the short-term but exact measurement by CGM, HbA1c variability had a different presentation of mean glucose variability over a longer period.

This pilot study, however, had several limitations. First, this study had a relatively small sample size. Second, the interval between the two CGM periods was not long enough to observe a changing trend in HbA1c levels. Third, no intervention was involved for the participants’ daily physical activity. Thus, the impact of exercise could not be estimated. Fourth, the glycemic index of food was not calculated. Fifth, although beta-cell failure can diminish the glycemic effect of GLP1-RA, data on c-peptide levels in this study were incomplete given that this study was retrospective and that c-peptide levels were not routinely examined. However, this study could serve as a pilot for future studies on new and improved antidiabetic regimens.

## Conclusions

In conclusion, GLP-1 RA, in combination with basal insulin, regimen can better stabilize GV than a twice-daily pre-mixed insulin regimen, especially in the daytime, although no significant impact on the consumption of macro-nutrition and calories was found.

## Supplementary Information

**Additional file 1: Table S1.** Correlation of MAGE, SD, CV and Age, BMI, diabetes duration, HbA1c value before CGM study, percentages of carbohydrate, protein, and fat intake per day.

## Data Availability

Not applicable.

## Abbreviations

HbA1c: Glycated hemoglobin A1c
GV: Glycemic variability
CGM: Continuous glucose monitoring
CHO: Carbohydrate
PRO: Protein
GLP-1: Glucagon-like peptide 1
RA: Receptor agonists
OAD: Oral antidiabetic drugs
CGMH: Chang Gung Memorial Hospital
SD: Standard deviation
CV: Coefficient of variation
MAGE: Mean amplitude of glucose excursion
TIR: Time in range
SMBG: Self-monitoring of blood glucose
AUC: Area under the curve
LBGI: Low blood glucose index
HBGI: High blood glucose index
CONGA: Continuous overlapping net glycemic action
