# Correction: Regimen comprising GLP‑1 receptor agonist and basal insulin can decrease the effect of food on glycemic variability compared to a pre‑mixed insulin regimen

**DOI:** 10.1186/s40001-024-02149-z

**Published:** 2024-11-28

**Authors:** Yi‑Hsuan Lin, Chia‑Hung Lin, Yu‑Yao Huang, Hsin‑Yun Chen, An‑Shun Tai, Shih‑Chen Fu, Sheng‑Hwu Hsieh, Jui‑Hung Sun, Szu‑Tah Chen, Sheng‑Hsuan Lin

**Affiliations:** 1 Division of Endocrinology and Metabolism, Department of Internal Medicine, Chang Gung Memorial Hospital, Linkou, Taiwan; 2 Department of Biological Science and Technology, National Yang Ming Chiao Tung University, Hsinchu, Taiwan; 3 Department of Chinese Medicine, College of Medicine, Chang Gung University, Taoyuan, Taiwan; 4 Department of Medical Nutrition Therapy, Chang Gung Memorial Hospital, Linkou, Taiwan; 5 Institute of Statistics, National Yang Ming Chiao Tung University, Hsinchu, Taiwan

**Correction: European Journal of Medical Research (2022) 27:273** 10.1186/s40001-022-00892-9.

In this article the affiliation details for Yi-Hsuan Lin was missing it should be “Department of Biological Science and Technology, National Yang Ming Chiao Tung University, Hsinchu, Taiwan” the affiliation details for the authors, An-Shun Tai, Shih-Chen Fu and Sheng-Hsuan Lin were incorrectly given as “Institute of Statistics, National Chiao Tung University, Hsinchu, Taiwan” But should have been Institute of Statistics, National Yang Ming Chiao Tung University, Hsinchu, Taiwan.
