# Supplementary material for: Regimen comprising GLP-1 receptor agonist and basal insulin can decrease the effect of food on glycemic variability compared to a pre-mixed insulin regimen
Source: Eur J Med Res. 2022 Dec 3;27:273. doi: 10.1186/s40001-022-00892-9 (PMC9719195; doi:10.1186/s40001-022-00892-9)
Supplement: Supplementary file 1 — Additional file 1: Table S1. Correlation of MAGE, SD, CV and Age, BMI, diabetes duration, HbA1c value before CGM study, percentages of carbohydrate, protein, and fat intake per day. [file 40001_2022_892_MOESM1_ESM.docx]

Supplement Table S1. Correlation of MAGE, SD, CV and Age, BMI, diabetes duration, HbA1c value before CGM study, percentages of carbohydrate, protein, and fat intake per day

| Parameter | MAGE (mg/dl) | | SD (mg/dl) | | CV (%) | |
| --- | --- | --- | --- | --- | --- | --- |
|  | Correlation coefficient | p value | Correlation coefficient | p value | Correlation coefficient | p value |
| Age (years) | 0.516 | 0.002* | 0.552 | 0.001* | 0.597 | <0.001* |
| BMI | -0.276 | 0.129 | -0.296 | 0.100 | -0.328 | 0.067 |
| Diabetes duration (years) | 0.407 | 0.021* | 0.439 | 0.012* | 0.443 | 0.011* |
| HbA1c (%, mmol/mol) | -0.138 | 0.458 | -0.089 | 0.635 | -0.387 | 0.031* |
| CHO (%) | 0.331 | 0.064 | 0.366 | 0.040* | 0.420 | 0.017* |
| Pro (%) | -0.214 | 0.240 | -0.311 | 0.084 | -0.423 | 0.016* |
| Fat (%) | -0.325 | 0.070 | -0.336 | 0.060 | -0.363 | 0.041* |

*Spearman’s Correlation is significant at the 0.05 level (2-tailed)

Abbreviation: MAGE: mean amplitude of glycemic excursions; SD: standard deviation; CV: coefficient of variation; BMI: body mass index; HbA1c: glycated hemoglobulin A1C; CGM: continuous glucose monitoring; CHO: carbohydrate; Pro: protein
